# Whole-body MRI for the evaluation of metastatic lobular breast cancer; current uses and future directions

**DOI:** 10.1093/bjro/tzag018

**Published:** 2026-09-15

**Authors:** Kate Downey, Basrull N Bhaludin, Nina Tunariu, Alicia F C Okines, Christina Messiou, Dow-Mu Koh

**Affiliations:** Department of Radiology, The Royal Marsden Hospital NHS Foundation Trust, London SW6 6JJ and Sutton SM2 5PT, United Kingdom; The Institute of Cancer Research, London SW6 6JB and Sutton SM2 5GP, United Kingdom; Department of Radiology, The Royal Marsden Hospital NHS Foundation Trust, London SW6 6JJ and Sutton SM2 5PT, United Kingdom; The Institute of Cancer Research, London SW6 6JB and Sutton SM2 5GP, United Kingdom; Department of Radiology, The Royal Marsden Hospital NHS Foundation Trust, London SW6 6JJ and Sutton SM2 5PT, United Kingdom; The Institute of Cancer Research, London SW6 6JB and Sutton SM2 5GP, United Kingdom; The Institute of Cancer Research, London SW6 6JB and Sutton SM2 5GP, United Kingdom; Department of Clinical Oncology, The Royal Marsden Hospital NHS Foundation Trust, London SW3 6JJ and Sutton SM2 5PT, United Kingdom; Department of Radiology, The Royal Marsden Hospital NHS Foundation Trust, London SW6 6JJ and Sutton SM2 5PT, United Kingdom; The Institute of Cancer Research, London SW6 6JB and Sutton SM2 5GP, United Kingdom; Department of Radiology, The Royal Marsden Hospital NHS Foundation Trust, London SW6 6JJ and Sutton SM2 5PT, United Kingdom; The Institute of Cancer Research, London SW6 6JB and Sutton SM2 5GP, United Kingdom

**Keywords:** metastatic lobular breast cancer, whole-body MRI, imaging

## Abstract

Invasive lobular cancer (ILC) metastases are harder to detect radiologically than invasive breast cancer of no specific type due to a unique tumor biology. Metastatic ILC (mILC) spread is also unusual and challenging, favoring sites such as the peritoneum. The sclerotic bone metastases commonly seen with mILC are non-measurable on CT by currently accepted response criteria (RECIST 1.1.) and are frequently fluorodeoxyglucose (^18^F-FDG) PET-CT non-avid. UK/European guidelines recommend CT +/− bone scan or PET-CT for staging and assessment of treatment response in breast cancer however there is increasing evidence that Whole-body MRI (WBMRI) with its anatomical and functional capabilities (offered by diffusion-weighted imaging) can overcome conventional imaging challenges for breast cancer, especially for ILC subtypes. WBMRI is more accurate at identifying additional sites of disease in mILC and the earlier detection of progressive disease resulting in treatment changes. WBMRI advancements are creating fast, well-tolerated studies, and the use of novel artificial intelligence and machine learning techniques may increasingly help with acquisition and reporting speed. There are WBMRI consensus guidelines for several tumor types, but no published guidelines yet exist for breast. This review article presents the current evidence to support the use of WBMRI in the management of mILC illustrated by cases from clinical practice and discusses the challenges of widespread adoption and potential future applications.

## Introduction

Breast cancer is the most common cancer in the UK and Europe and the leading cause of death in females.^1^ The incidence is rising rapidly; since the early 1990s, rates of breast cancer have increased by approximately a quarter in the UK.^2^ Lobular breast cancers originate in the lobules (milk producing glands) of the breast and are the second most common subtype of invasive breast cancer accounting for up to 15% of new diagnoses, second to invasive breast cancer of no specific type (IBC-NST—formally known as invasive ductal carcinoma). As it is harder to detect, invasive lobular cancer (ILC) can present at a later stage than IBC-NST and is more commonly multifocal.^3^ A recent large retrospective analysis found that ILC tumors were of lower grade but of a larger size at diagnosis with 14.3% ILC tumors stage T3/4 at presentation compared to only 3.4% of IBC-NST tumors.^4^ The diffuse infiltrative rather than mass-like growth pattern seen in ILC is due to the loss of E-cadherin (a protein responsible for cell adhesion encoded by the *CDH1* gene) creating a characteristic single cell pattern of stromal spread with relatively little desmoplastic reaction and architectural disruption of the tissues.^5^ This unique pattern of spread makes metastatic ILC (mILC) harder to detect and evaluate both clinically and radiologically than IBC-NST. mILC also favors sites that are distinct and challenging; often better suited to imaging with whole-body MRI (WBMRI) however current imaging guidelines recommend the same imaging for all breast cancer subtypes.

Whilst both ILC and IBC-NST metastases spread to the lymph nodes and liver, ILC more commonly spreads to the bones, peritoneum, ovaries, retroperitoneum, gastrointestinal (GI), genitourinary (GU) tract, and leptomeninges than IBC-NST.^3^^,^^6–14^ Parenchymal brain metastases are more commonly associated with triple negative and HER2 positive tumors than luminal hormone receptor positive ILC and IBC-NST tumors and lung metastases are more commonly seen with metastatic IBC-NST (mIBC-NST) than in mILC. mILC can also present with complications of metastatic disease such as hydronephrosis and bowel obstruction.^13^^,^^14^ Metastatic patterns of infiltrating lobular carcinoma metastases described in the literature are summarized in Table 1.

**Table 1:** Metastatic patterns of infiltrating lobular carcinoma metastases.

Reference	Numbers of breast cancer patients	Summary findings
**Harris et al Br J Cancer (1984)^6^**	Retrospective review 966 clinical cases (135 ILC) and 92 autopsy cases (14 ILC)	Peritoneal/retroperitoneal (93% vs 8% *P* < 0.0001), leptomeningeal (29% vs 1.3% *P* < 0.004), GI with infiltration of the stomach (43% vs 2.6% *P* < 0.0002) and bowel (29% vs 4% *P* < 0.02), GU (with diffuse infiltration of the uterus (43% vs 0% *P* < 0.0001), and ovary (36% vs 2.6% *P* < 0.0001) all more common with ILC vs IBC-NST at autopsy Involvement of the peritoneum/retroperitoneum was often associated with hydronephrosis (62%) No significant difference in rates of metastases between the tumor types for liver, bone, and pleural sites (although bone trephine more likely positive with ILC 72.2% vs 27.3% *P* < 0.0004). Lung and brain (parenchyma) metastases more common with IBC-NST
**Lamovec et al J Surg Oncol. (1991)^7^**	Retrospective review 261 Autopsy cases who died with metastatic breast cancer (25 ILC)	Peritoneal/retroperitoneal, GI, GU, leptomeninges, and pericardium more common sites in mILC (*P* < 0.006). Lung parenchymal metastases more common with IBC-NST vs ILC (*P* < 0.006) No statistical difference in frequency of spread to liver, bone, or pleura
**Dixon et al Br J Cancer (1991)^8^**	Retrospective review 149 clinical cases (77 ILC)	Peritoneal and liver metastases more common in ILC vs IBC-NST (*P =* 0.01 and 0.0003, respectively) No difference in the frequency of meningeal and pulmonary metastases between the 2 groups
**Borst et al Surgery (1993)^9^**	Retrospective review 2605 clinical cases (359 ILC)	Rates of metastases to GI system (4.5% vs 0.2%), gynecological organs (4.5% vs 0.8%), peritoneum/retroperitoneum (3.1% vs 0.6%), and bone marrow (21.2% vs 14.4%) were higher in ILC vs IBC-NST (*P* < 0.05) Rates of metastases to the lung/pleura (2.5% vs 10.2%) were significantly lower in ILC vs IBC-NST (*P* < 0.05) There was no significant difference in rates of metastatic disease to liver and CNS in this cohort
**Jain et al Eur J Cancer (1993)^10^**	Retrospective review 1391 clinical cases (177 ILC)	Peritoneal (11% vs 3%) and bone marrow (15% vs 5%) metastases more common in ILC vs IBC-NST (*P* < 0.01) Lung (31% vs 16%), pleura (34% vs 21%), and brain parenchyma (11% vs 2%) metastases more common in IBC-NST vs ILC (*P* < 0.05) Bone was the initial site of metastatic recurrence more frequently in ILC vs IBC-NST (52% vs 34% *P* < 0.01)
**Ferlicot et al Eur J Cancer (2004)^11^**	Retrospective review of anatomical location of first metastatic site/s from clinical cases 2845 (96 ILC)	Bone metastases more common in ILC vs IBC-NST (50% vs 38.5% *P* = 0.02) and other sites including peritoneum, GI tract, GU tract, and meninges (34.4% vs 9.9% *P* < 0.001) There was no difference in the rate of liver metastases. The frequency of lung and brain metastases were more common in IBC-NST vs ILC, but this did not reach significance
**Pestalozzi et al J Clin Oncol (2008)^3^**	Retrospective review 12 206 clinical cases from 15 breast cancer trials (767 ILC)	Bone metastases more common in ILC vs IBC-NST (*P* < 0.01) Lung metastases less common in ILC vs IBC-NST (*P* < 0.01)
**Inoue et al Breast Cancer (2017)^12^**	Retrospective review *N* = 330 clinical cases (19 ILC)	Frequency of peritoneal metastases significantly higher in ILC vs IBC-NST group (68.8% vs 1% *P =* 0.00). Frequency of lung metastases significantly lower in the ILC vs IBC-NST group (6.3% vs 53.7% *P* < 0.01). The prognosis after diagnosis of peritoneal metastases was poor (median survival time 19 ± 9 months)
**DiPiro et al Abdom Radiol (2019)^13^**	Retrospective review of patterns of intra-abdominal metastatic spread in ILC. *N* = 327 clinical cases	Most frequent sites of intra-abdominal metastatic sites were the peritoneum (47%), liver (32%), bowel (29%), ovary (28%), retroperitoneum (14%), ureter (14%), and lymph nodes (13%). Bowel obstruction in 15 patients (13%) and hydronephrosis in 25 patients (22%) Patients with abdominal metastases had shorter OS

Contemporary WBMRI studies combine different sequences that provide both excellent anatomical soft tissue contrast but also functional information from the diffusion-weighted imaging (DWI) sequences to interrogate differences in cellular density and water mobility between benign and malignant tissues without the need for intravenous (IV) contrast or ionizing radiation. WBMRI is well tolerated by most patients and takes 45-60 min to complete depending on the scanner type and protocol used.^15^ Although prospective studies are lacking, there is now increasing evidence that WBMRI can detect additional sites of disease in metastatic breast cancer compared with conventional imaging and can identify progressive disease (PD) at an earlier time point on treatment.^16–20^ Studies that divided the diagnostic performance by tumor subtype have demonstrated that using WBMRI for patients with mILC has a more significant impact on management decisions when compared to mIBC-NST.^16^^,^^18^^,^^20^ A summary of the main findings within the retrospective and prospective studies comparing WBMRI to other imaging modalities in breast cancer is given in Table 2.

**Table 2:** A summary of the main findings within the retrospective and prospective studies comparing WBMRI to other imaging modalities in breast cancer.

Reference	Cohort	Summary findings
**Bhaludin et al Cancer Imaging (2022)^16^**	Retrospective study Comparing WBMRI to CT TAP, bone scan, and FDG PET-CT for patients with metastatic breast cancer. *N* = 105 patients (ILC). 148 WBMRI studies compared to a control examination	50 pairs (33.8%) showed differences in the extent of disease, with 44 pairs due to additional sites (ASs) reported on WBMRI alone Group 1 (81 patients) had 1 WBMRI paired with routine imaging Group 2 (24 patients) had more than 1 WBMRI study paired with routine imaging (67 pairs) 13/67 pairs (19.4%) in Group 2 had discordant imaging findings with 10/13 pairs (76.9%) with progressive disease (PD) reported on WBMRI alone Treatment changes due to AS reported on WBMRI alone occurred in 21/23 pairs (91.3%) in Group 1 Treatment changes due to PD reported on WBMRI alone in Group 2 occurred in 6/14 pairs (42.9%) Treatment changes due to AS/PD in both the groups occurred in 11/102 pairs (10.8%) with NST vs 13/28 pairs (46.4%) with ILC
**Kosmin et al Radiology (2020)^17^**	Prospective study. Bone-only metastatic breast cancer (NCT03266744) comparing WBMRI to CT and BS. *N* = 45 (ILC *N* = 8).	WBMRI identified PD before CT with bone only metastatic breast cancer. PD evident on WBMRI in 67% and on CT in only 33% concurrently; none had PD on CT only (*P* < 0.01). PD on BS was reported in only 50% (*P* < 0.01)
**Zugni et al PLoS One (2018)^18^**	Retrospective study. Comparing WBMRI to CT TAP and FDG PET-CT for patients with metastatic breast cancer. *N* = 58 patients (ILC 19). 910 WBMRI studies (58 compared to control imaging study)	In 23/58 (39.6%) paired examinations, ASs of disease were reported on WB-MRI only and not on control studies In 17/28 (60.7%) paired examinations, PD reported on WB-MRI only and not on control studies In 14/28 (50%) paired examinations that were followed by a change in treatment, PD had been reported on WBMRI only, while stable disease (SD) had been reported on the control study Inside the subgroup of 19 paired examinations in patients with ILC, therapy was changed because of PD in 10/19 (52.9%) patients and PD reported only on WBMRI alone in 7/10 (70%) patients. The most frequent AS of disease in the ILC subgroup was bone and second was the peritoneum/retroperitoneum
**Kosmin et al Eur J Cancer (2017)^19^**	Retrospective study. Comparing WBMRI to CT TAP for patients with metastatic breast cancer. *N* = 101 patients. 210 pairs of WBMRI and CT	WBMRI reported ASs of disease not reported on CT in 53.3% of episodes Discordant imaging findings at response assessment were found in 28% of cases; most commonly PD found on WBMRI being reported as SD on CT (18.9%) Discordant response assessments were less common in first line treatment regimens than on subsequent treatment. PD was reported on WBMRI only in 34.7% of episodes where treatment was changed

The management of mILC is becoming increasingly complex and personalized and a multidisciplinary team approach is required for optimized patient outcomes. With increasing understanding of the molecular and genetic profile heterogeneity of ILC variants, the treatment options for patients with mILC are widening. Treatment includes endocrine therapy, chemotherapy, and more recently immunotherapy (the latter for rare triple negative cases) either consecutively or in combination. Focal radiotherapy can also be used for oligometastatic disease (OMD) and palliative symptom control. Patients diagnosed with metastases are referred for an image-guided biopsy to confirm histology and to assess estrogen receptor (ER), progesterone receptor (PR), and human epidermal growth factor receptor (HER2) status to guide treatment. As the majority of ILC tumors are ER and PR positive (ER+/PR+) and HER2 negative (HER2−), endocrine-based therapy is commonly used as first line, normally in combination with other treatments. Improvements in both progression-free survival (PFS) and overall survival (OS) are seen with the addition of CDK4/6 inhibitors to endocrine therapy in ER+/HER2− disease.^21^ The use of anti-HER2 therapy is recommended in rare cases of HER2 positivity. Mutations in *ERBB2*, *AKT1*, and *PIK3CA* are all enriched in mILC, whereas mutations in *ESR1* occur at a similar frequency to IBC-NST.^22^ A minority of mILC tumors are associated with germline mutations (most commonly *BRCA2*) which sensitizes cancers to targeted agents such as PARP inhibitors. With increasing molecular profiling of these mutations and understanding of their interactions, targeted therapies are viable treatment options for many patients diagnosed with mILC.^23^

The limitations of conventional imaging techniques in detecting and evaluating the extent of metastatic disease in lobular breast cancer and assessing the response to treatment have come into focus in recent years. WBMRI can detect additional sites of mILC that are challenging using conventional imaging which may reduce late presentation. Local treatment of OMD (commonly defined as up to 5 solid metastatic sites) can significantly prolong PFS, but no significant overall survival benefit has been demonstrated thus far.^24^ Successful treatment of OMD depends on the accurate identification of the number and location of disease sites. The most recent international consensus guidelines recommend either ^18^F-FDG PET-CT or WBMRI for the confirmation of OMD in breast cancer and state that different tumor subtypes may require dedicated imaging strategies referencing the 2022 paper from Bhaludin et al on the added value of WBMRI in lobular breast cancer.^20^^,^^25^ Lastly, WBMRI is more accurate in defining disease status in breast cancer patients who have had multiple lines of treatment.^19^ This is likely due to increasing disease heterogeneity and imaging complexity with increasing lines of treatment, with WBMRI capable of defining a combination of treated and active disease.

## WBMRI protocol and technical considerations

The WBMRI protocol used at The Royal Marsden Hospital (RMH) for the assessment of breast cancer spread most closely aligns with that recommended in the MET-Rads-P WBMRI consensus guidelines and is similar to those recommended by MY-Rads and ONCO-RADS which are all shown to have a high diagnostic performance in prostate cancers, myeloma, and cancer screening, respectively.^26–28^ The protocols are available to download.^29^ The protocol can be performed at either 1.5 T (preferred) or 3 T and includes (i) anatomical T1Weighted (W) and T2W TSE sagittal spine imaging (4-5 mm slice thickness), and matched axial body (cover vertex to thighs) (ii) T2W HASTE, (iii) T1W GRE DIXON (in and out-of-phase, water-only, and fat-only sequences and derived fat and water fraction maps), (iv) diffusion-weighted STIR fat-suppressed with 3b-values (b-50, 600, and 900 s/mm^2^) and an apparent diffusion coefficient (ADC) map from mono-exponential data fitting, (iv) axial FLAIR images through the brain (vertex to skull base) to screen for asymptomatic large brain metastases (>1 cm). All axial sequences are 5 mm in slice thickness, with no gaps to allow the use of WBMRI protocol for RECIST purposes. A maximum intensity projection (MIP) image is reconstructed from the b-900 diffusion-weighted sequences and can be useful in visualizing overall disease burden. An additional post contrast 3D T1W sequence through the brain is added in patients with known brain metastases.

Diffusion-weighted imaging (DWI) is an established functional imaging technique used in WBMRI with the ability to distinguish benign from malignant tissues by providing information related to cellular density and the ease of water diffusivity within tissues. It uses a quantitative parameter/surrogate imaging biomarker, the ADC and is a well-established technique in oncological imaging. A UK quantitative WBMRI working group published guidelines in 2018 for the use of DWI within the WBMRI setting to ensure accuracy and reproducibility.^30^ More recently the Quantitative Medical Imaging Coalition (QMIC) has opened a new profile for “Whole-body DWI for tumor burden and ADC quantification in oncologic bone disease” which will be an important step forward for standardized quantitative WBMRI.^31^ Prior studies have established ADC values in prostate cancer to be repeatable and MET-RADS-P describes threshold ADC values of normal bone marrow, viable tumor cells, and areas of response to treatment within the skeletal metastases.^26^^,^^32^ Areas of viable tumor appear hyperintense on high b-value (b-900) images, have low ADC values, and correlate with lesions on anatomical T1W and T2W sequences. Benign conditions such as fractures with bone marrow edema and normal structures such as the bowel, ovaries, and lymph nodes can all demonstrate high signal on high b-value images due to T2 shine through. It is therefore essential to compare the high b-value images with the low b-value images, the ADC map, and the other anatomical sequences to distinguish between normal structures and disease.

The relative fat-fraction (rFF) map is a post-processed set of images derived from T1W DIXON sequences which produces 4 sets of images from 1 data set acquisition: in-phase, out-of-phase, fat-only, and water-only images. The rFF is the fat content percentage in each voxel of the image produced and is calculated by removing the signal from the water only images from the in-phase images and dividing the result by the in-phase voxel signal. The resulting calculation is displayed as a “rFF map” matched to the other axial sequences. Analogous calculations can be used to create a relative water fraction (rWF) map. Malignant bone marrow infiltration replaces the normal bone marrow fat and therefore demonstrates low signal against the high signal surrounding fat within the bone marrow and corresponding high signal on the water fraction due to the increased water content of tumors and tissue perfusion (Figure 1). The DIXON method and the derived fat- and water-fraction maps can distinguish between benign and malignant disease and allow the quantitative assessment of the fat within the bone marrow before and after treatment. Malignant bone lesions in breast and prostate cancer and myeloma show significantly lower median rFF compared with normal bone marrow with excellent interobserver agreement and demonstrate no significant alteration in measurements according to tumor origin.^33^ rFF images have also been found to have a higher relative contrast ratio compared with T1W and high b-value (b-900) DWI.^34^ The mean ADC and rFF percentages of single lesions as well as global disease volumes have been found to be repeatable measurements in prostate cancer skeletal metastases showing potential as quantitative biomarkers in metastatic bone disease and response assessments.^32^^,^^35^ ADC and rFF values are being established as robust imaging biomarkers in the evaluation of metastatic disease but at present they remain at the investigational stage for breast cancer and mILC. The ADC value used in Figure 2 in this review should be regarded as illustrative and its interpretation has been extrapolated from work in other tumor types. However, whilst no prospectively validated threshold values exist for metastatic breast cancer, published data confirms that the ADC of bone metastases reflects tumor cellularity regardless of the primary tumor site.^36^^,^^37^

**Figure 1:**
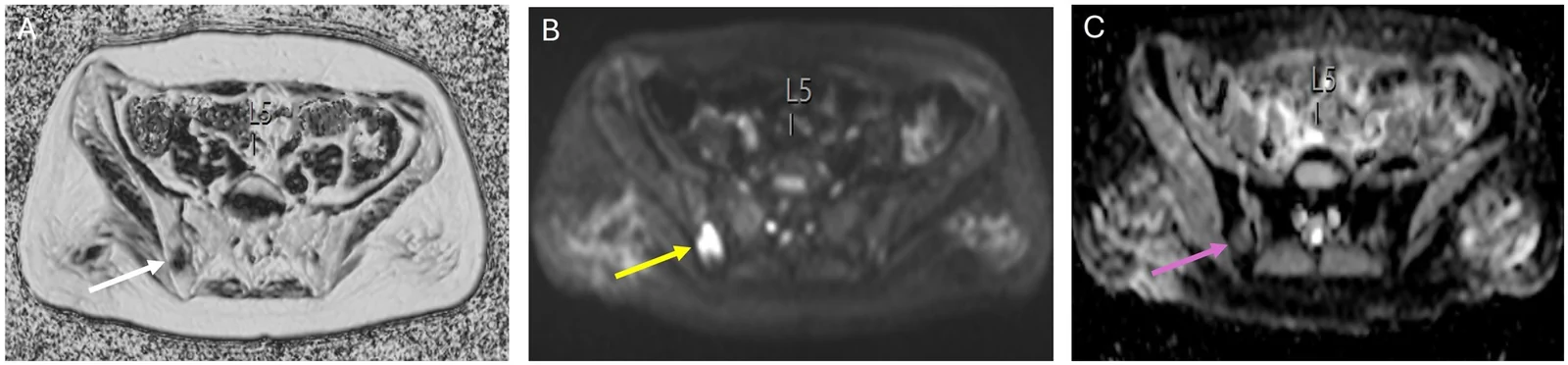
Biopsy proven lobular carcinoma of the right breast (ER+, PR+, and HER2− grade 2 ILC) following symptomatic presentation with de novo skeletal metastatic disease. A site of active metastatic disease within the right iliac bone on WBMRI replaces the normal fat of the bone marrow with an area of low signal on the rFF map (A) and is seen as an area of high signal on the DWI sequences (b-900) (B) with a corresponding focus of low signal on the ADC map (C). Please note that the ADC of normal bone is intrinsically low owing to the high fat content of the bone marrow; consequently, tumor generally exhibits a higher ADC than the adjacent normal bone.

**Figure 2:**
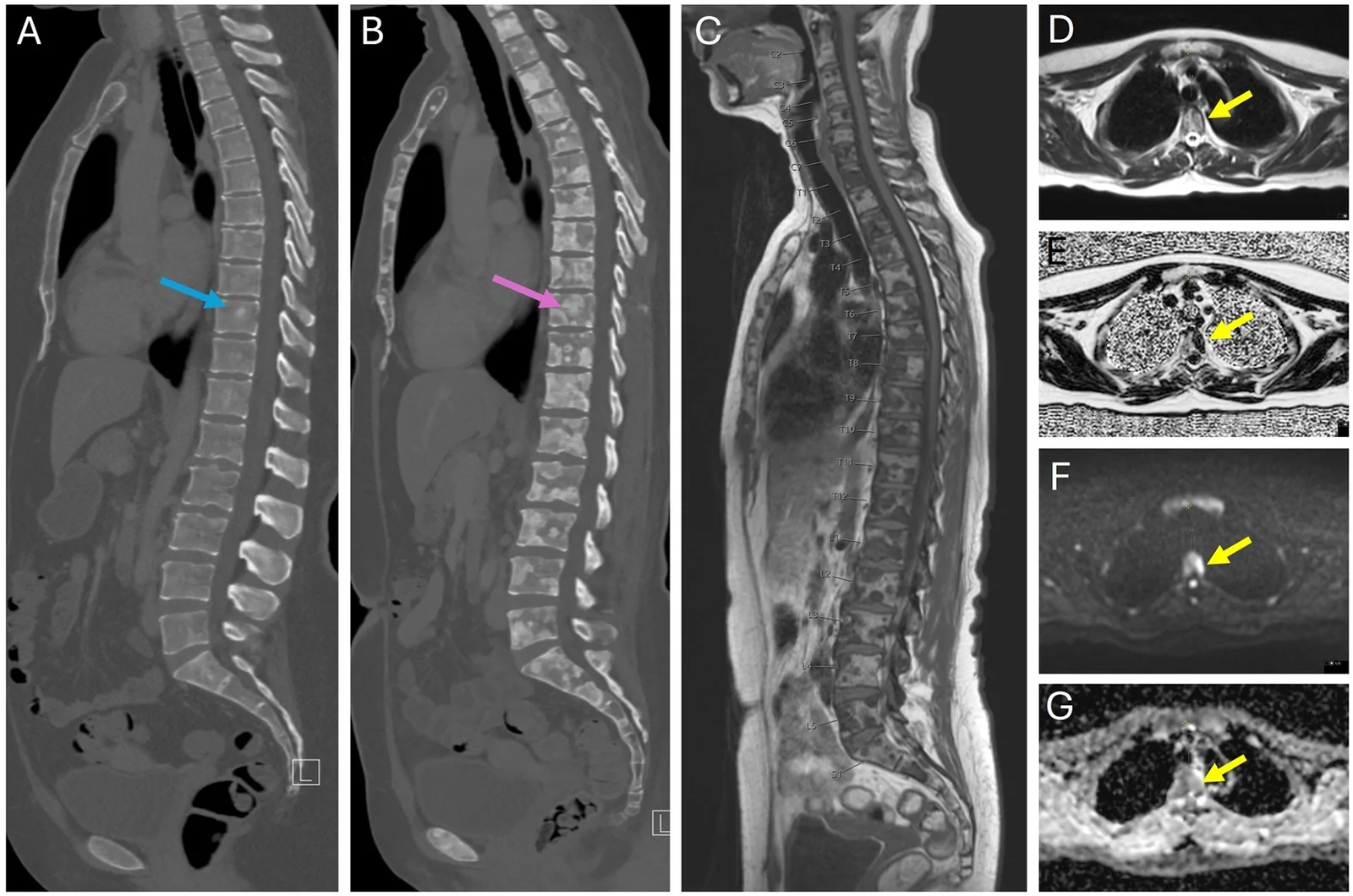
Biopsy proven symptomatic right breast cancer with axillary lymph node spread and *de novo* skeletal metastases (ER+, PR+, and HER2− grade 2 ILC). CT at diagnosis for staging (A) demonstrates small volume sclerotic skeletal foci. Systemic treatment was commenced, and interval CT (B) demonstrates an increase in size of the T10 lesion and an indeterminate increase in the number of sclerotic skeletal metastases throughout. Contemporaneous WBMRI confirms widespread disease on the anatomical T1W sagittal (C) and axial T2W (D) images but signal characteristics in keeping with treated disease therefore a response to treatment. The fat of T4 vertebral body is infiltrated by metastatic disease on the relative fat fraction (rFF) map (E) and the DWI demonstrates high signal on the high b-value image (b-900) (G) but relatively high signal on the ADC map (ADC 1100 mm^2^/s) (I) in keeping with low cellularity treated disease.

**Figure 3:**
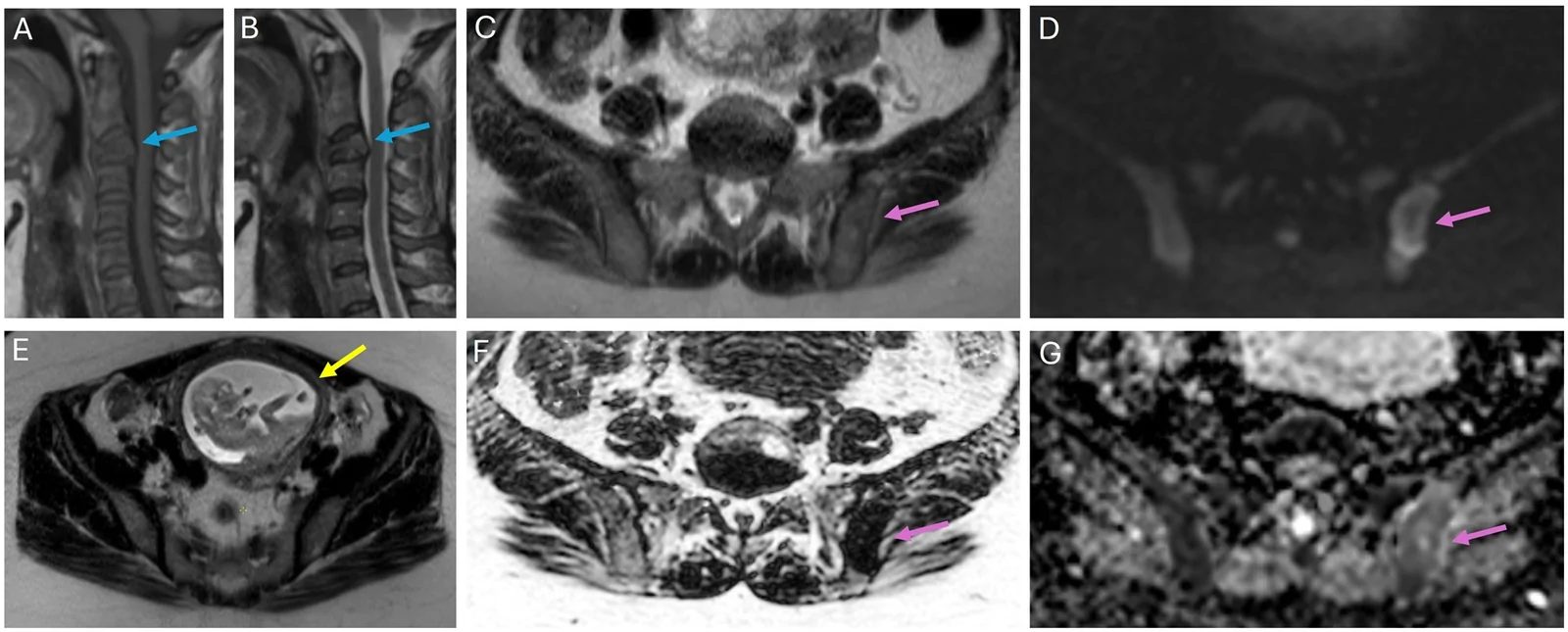
Biopsy proven symptomatic right breast cancer in pregnancy (ER+, PR+, and HER2− grade 2 ILC). WBMRI demonstrates a skeletal metastasis at C3 on the sagittal T1W (A) and T2W (B) imaging with a pathological fracture. The study also demonstrates a left iliac bone metastasis; subtle on the axial T2W sequence (C) but clearly seen on the DWI from the WBMRI study with high signal on the b-900 image (D) and low signal on the ADC map (G). It replaces the normal bone marrow fat on the rFF map (F). The intrauterine pregnancy is noted (E).

**Figure 4:**
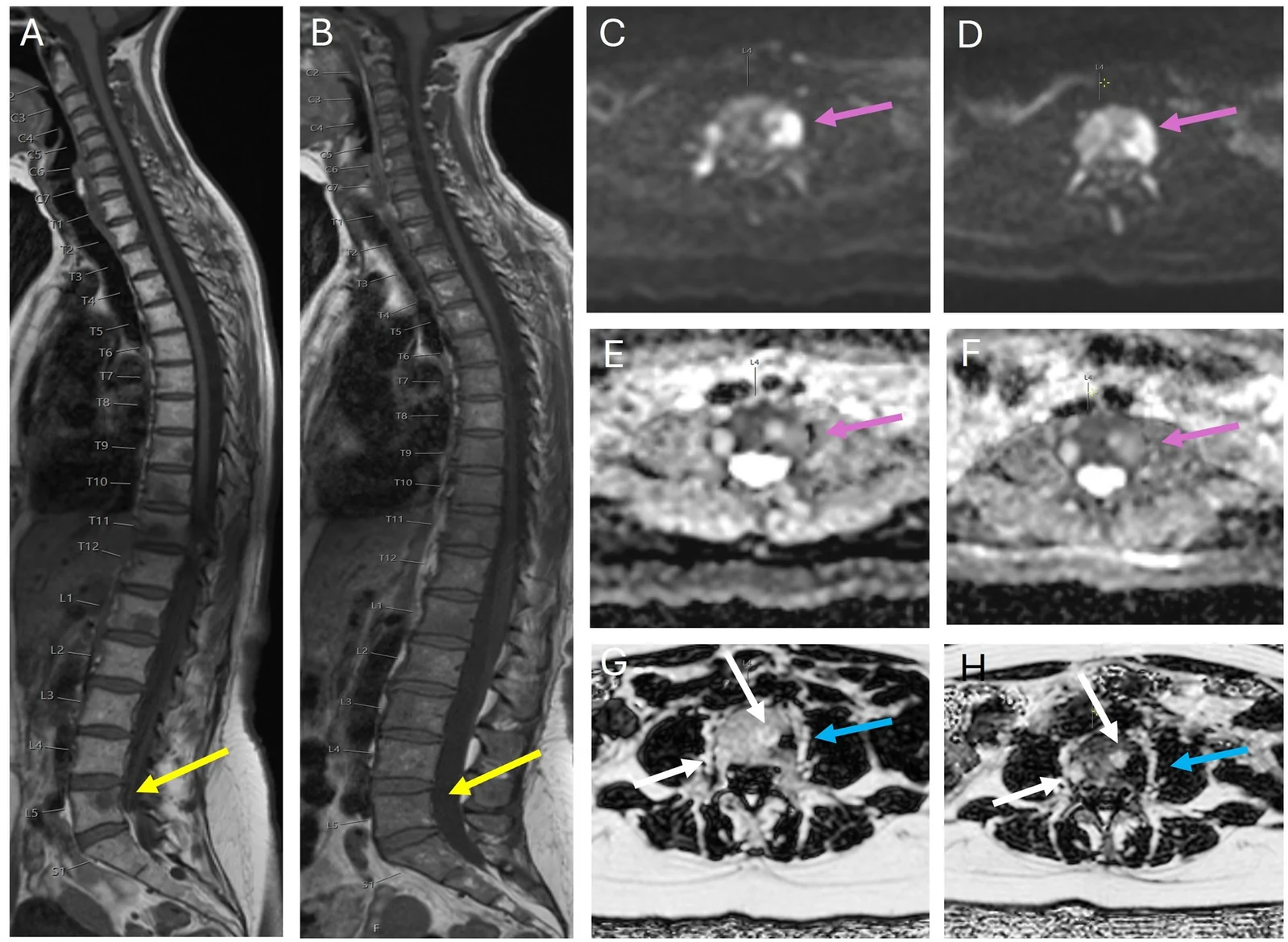
Biopsy proven lobular carcinoma of the breast (ER+, PR−, and HER2− grade 2 ILC). Metastatic recurrence with skeletal disease following initial treatment for node-positive disease. Two WBMRI studies were performed (baseline and at 8 weeks post-treatment). The patient was given granulocyte colony stimulating factor (GCSF) for bone marrow support during treatment. The sagittal T1W sequence (A) from the baseline WBMRI demonstrates multiple low signal skeletal metastases (eg, L5 lesion). This is much harder to see on the later sagittal T1W sequence (B) post-GCSF. A further L4 lesion demonstrates a small interval increase in size on the DWI (C-F) and whilst the contrast remains high between the tumor and the normal bone marrow on the DWI on both the earlier pre-GSCF (C and E) and post-GCSF (D and F) and whilst the lesion is easy to see on the rFF map pre-GCSF (G), contrast is reduced on the rFF map between the tumor and the background normal bone marrow on the post-GCSF image (H). Of note the benign fat containing vertebral hemangiomas are also more conspicuous against the more cellular background marrow following GCSF (H).

**Figure 5:**
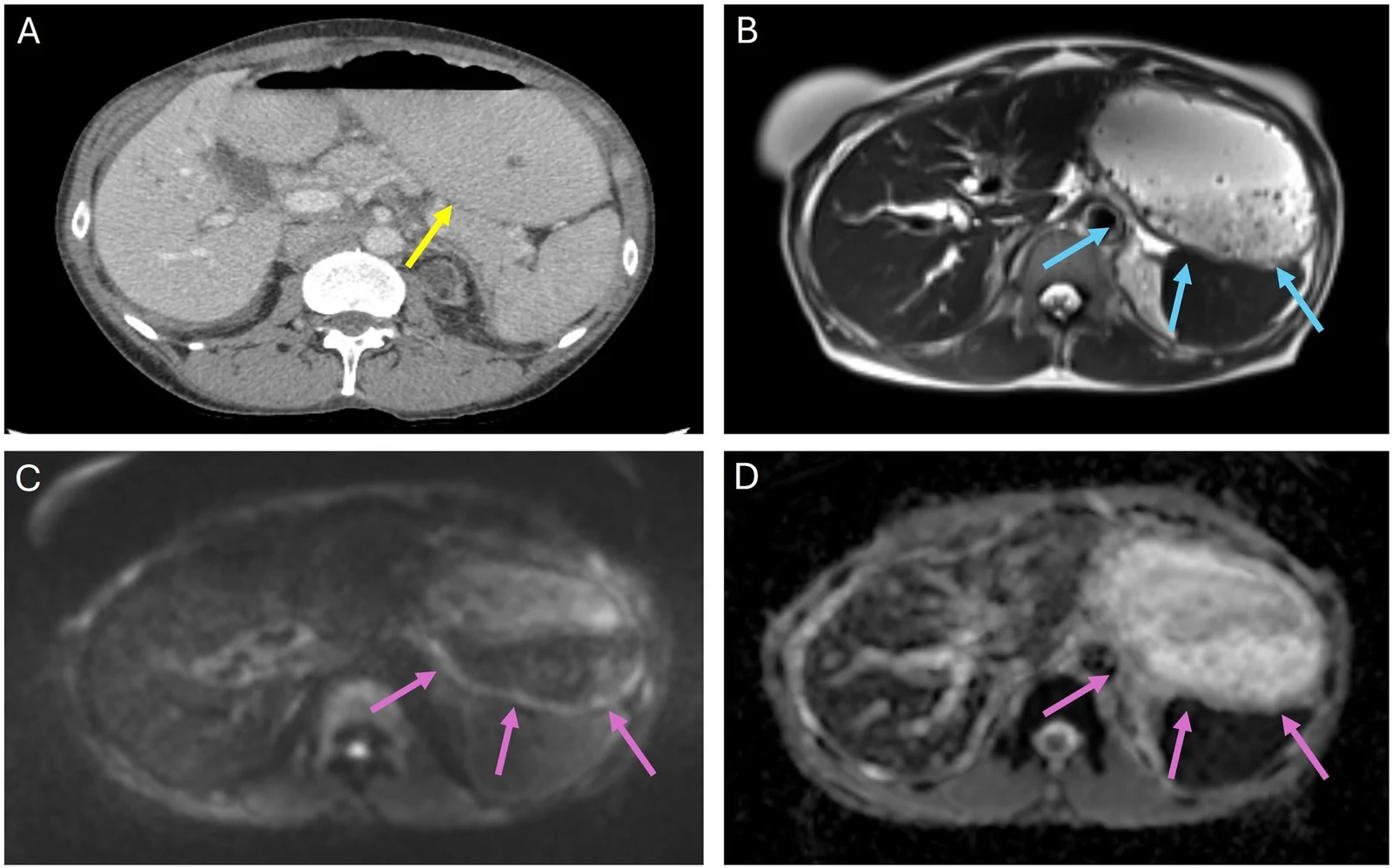
Biopsy proven ILC (ER+, PR+, and HER2− grade 2) of the breast treated with mastectomy with axillary clearance (positive lymph nodes) and adjuvant chemotherapy. Presentation with abdominal symptoms of indigestion and cramping abdominopelvic pain 8 years later. CT (A) demonstrates a distended stomach (arrow) but no obstructing mass. Gastroscopy demonstrates no mass lesion, but a biopsy sample is infiltrated by malignant cells consistent with invasive lobular cancer. The stomach appears normal on the axial T2W image (B) from the WBMRI, but there is subtle thickening and corresponding abnormal signal on the DWI sequences with high signal on the high b-value image (b-900) (C) and relatively low signal on the ADC map (D) in keeping with the biopsy proven disease.

**Figure 6:**
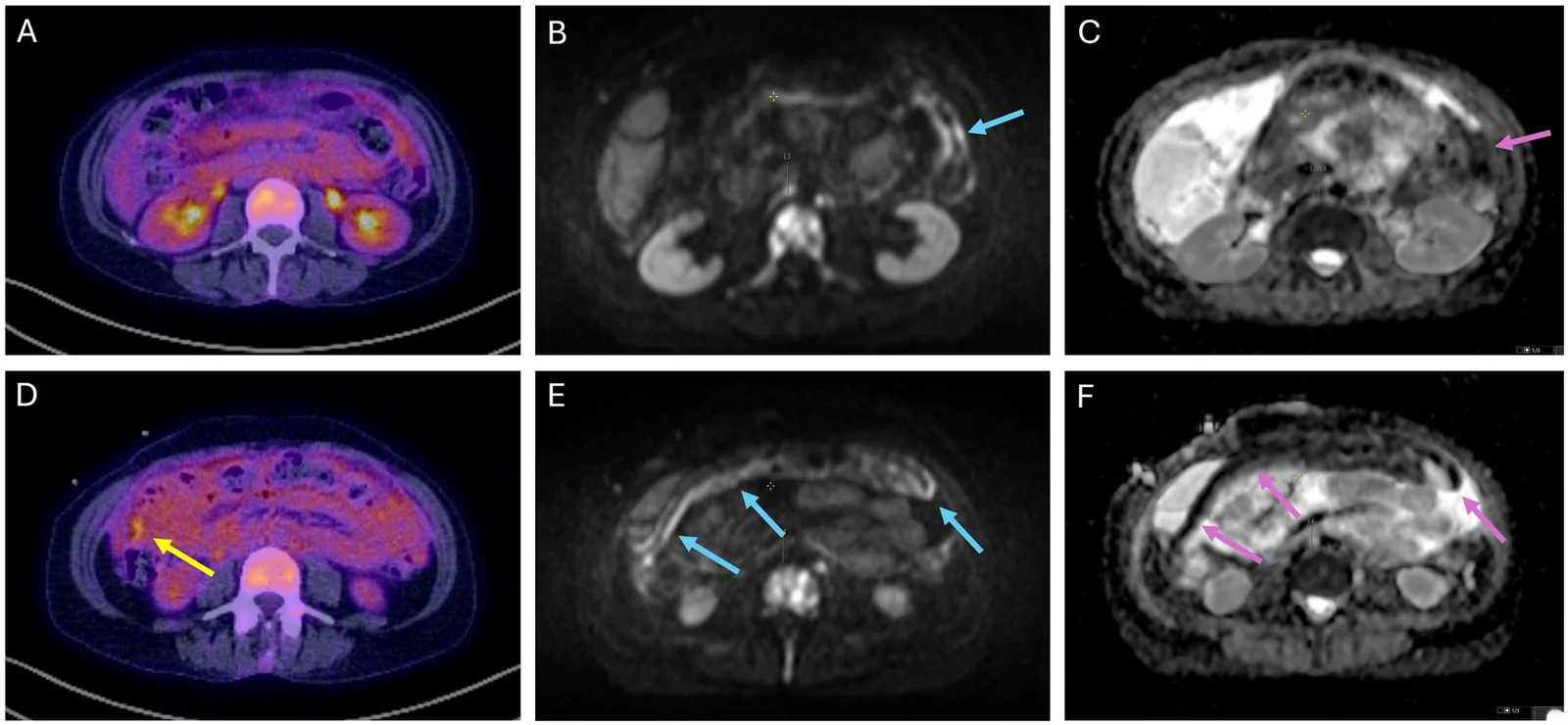
Biopsy proven lobular carcinoma of the breast (ER+, PR+, and HER2− grade 2 mixed ductal and lobular) following symptomatic presentation. Metastatic relapse at 5 years post-treatment with peritoneal and bone disease. The ^18^F-FDG PET-CT demonstrates small volume peritoneal disease significantly underestimated when compared to the WBMRI performed to further evaluate clinical findings. PET-CT demonstrates no significant uptake in the left flank (A) however WBMRI demonstrates peritoneal disease in this location with soft tissue thickening on the T2W sequences and corresponding high signal on the b-900 image from the DWI sequences (B) and corresponding low signal on the ADC map (C) suggesting high cellularity active disease. In the same patient, there is low grade uptake in keeping with peritoneal disease within the right abdomen on PET-CT (D) however WBMRI demonstrates much more extensive disease related to the omentum with high signal on the b-900 image from the DWI sequences (E) and corresponding low signal on the ADC map (F).

**Figure 7:**
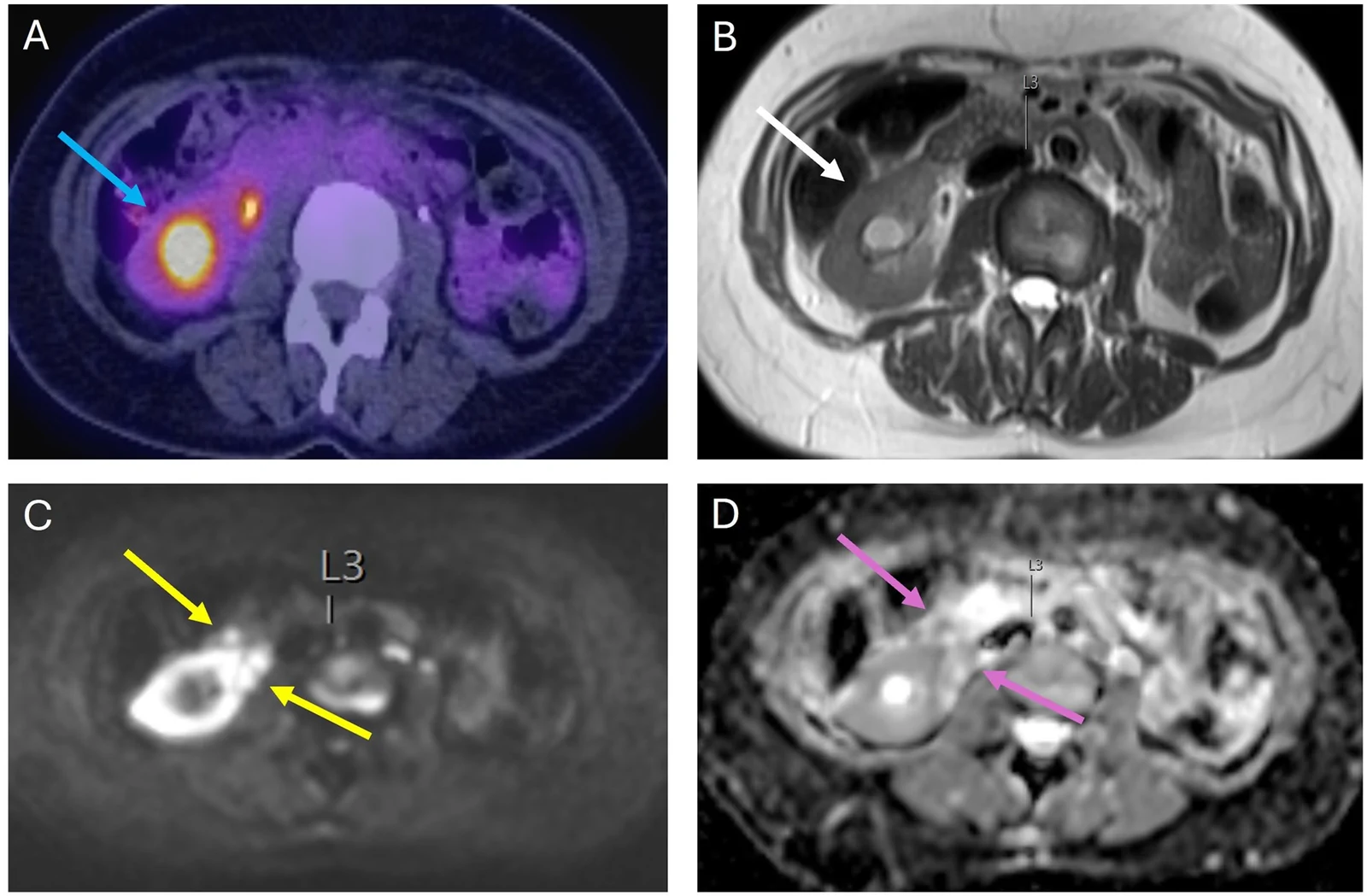
Biopsy proven lobular carcinoma of the right breast following symptomatic presentation with breast changes (ER+, PR+, and HER2− grade 2 ILC) with *de novo* skeletal metastatic disease demonstrated on staging ^18^F-FDG PET-CT. Follow-up PET-CT following treatment demonstrates a complete metabolic response. Four years later the patient presents with back pain. PET-CT (A) demonstrates new hydronephrosis. Contemporaneous WBMRI performed for further evaluation also demonstrates right sided hydronephrosis on the axial T2W sequences (B) but also disease infiltrating the right renal capsule seen as high signal nodules on the b-900 images from the DWI sequences (C) and corresponding low signal nodules on the ADC map (D) confirming PET-CT non-avid genitourinary metastatic disease. Of note, the disease was FDG-avid on PET-CT at diagnosis but significantly underestimated the extent of disease at time of relapse when compared to the WBMRI performed at the same time point.

## Current imaging guidelines for staging and response assessment in mILC and the role of WBMRI

Staging with contrast enhanced CT of the thorax, abdomen, and pelvis (CT-TAP) with or without BS is currently recommended for all breast cancer subtypes in patients with large tumors over 5 cm (T3) or where there is local infiltration of adjacent structures such as the chest wall or skin (T4), for patients who have 4 or more abnormal lymph nodes on ultrasound assessment or 4 or more macro-metastases at time of axillary surgery and is commonly requested for node-positive patients embarking on neoadjuvant chemotherapy. Staging is also recommended for patients who have symptoms of metastatic disease or biopsy proven recurrence.^38^

When patients are diagnosed with metastatic disease, imaging is usually performed at baseline and then to assess response at intervals during treatment, often every 3 months but this can be more frequent depending on the site of metastatic disease, patient’s symptoms, laboratory findings, and treatment regimen, or less frequent for patients experiencing a prolonged benefit. Current UK guidelines recommend CT for initial assessment whilst ^18^F-FDG PET-CT is preserved for inflammatory breast cancers and problem solving. WBMRI is now within the UK guidelines for breast cancer for staging women diagnosed with breast cancer in pregnancy and for problem solving when conventional imaging is equivocal regardless of tumor subtype (Figure 3). The recently released 2025 RCR guidelines now state that WBMRI may be utilized for baseline staging which is a new addition when compared to the 2019 Fourth edition.^38^

CT is currently recommended within both national and international guidelines for staging and response assessment for all metastatic breast cancer subtypes.^38–40^ It is fast, widely available, reproducible, standardized, and commonly used in clinical practice. Imaging of the chest, abdomen, and pelvis is performed after IV contrast administration, unless there is a contraindication, with coverage usually from the supraclavicular fossae to the proximal femora. The study requires IV access and uses ionizing radiation. Accurate staging and response assessment in the presence of bone only, bone predominant, peritoneal & retroperitoneal disease, and liver pseudocirrhosis (usually due to scarring post-treatment) are amongst some of the biggest limitations when using CT.

Bone metastases are the most common metastatic site in mILC and seen more frequently in lobular subtypes than IBC-NST subtypes.^3^^,^^6^ It is well recognized that CT is less accurate when compared with WBMRI in the assessment of response within metastatic bone disease in breast cancer.^41^ Skeletal disease in mILC is most often sclerotic but can be difficult to identify or even CT occult. The standardized response evaluation criteria (RECIST 1.1) require the presence of lytic bone disease with an identifiable soft tissue component over 1 cm in size.^42^ Sclerotic lesions are not considered evaluable and patients who do not meet RECIST 1.1 criteria are often excluded from clinical trials. There are no recognized CT criteria for assessing sclerotic bone lesions as CT cannot differentiate between sclerotic response and sclerotic progression. An increase in sclerosis or the development of new sclerotic lesions during treatment on CT can either represent progression or a healing response to therapy.

Whole-body MRI can distinguish benign from malignant bone lesions, as well as characterize treatment response by identifying and measuring the tissue cellularity and bone marrow replacement using functional DWI and derived fat-fraction images (rFF maps) from the DIXON sequences, thus offering new promising criteria for response in bone.^26^ Studies that have compared WBMRI to conventional imaging in metastatic breast cancer have found that there are consistent differences in response evaluations with WBMRI detecting PD and systemic therapy change at an earlier time point when compared to CT and PET-CT. PD within the skeleton has been reported only on WBMRI with a failure to identify advancing disease on CT, PET-CT, and bone scan (BS) in up to 70% of patients with ILC.^17^^,^^18^

For accurate interpretation of the extent of skeletal disease on WBMRI it is important to know the patient history and timing of the baseline imaging in relation to the start of anti-cancer treatment, the tumor marker trend and whether the patient is receiving medication for neutropenia. The bone marrow stimulation that results from granulocyte colony stimulating factor (GCSF) administration can mimic diffuse bone marrow disease within as little as 2 weeks (Figure 4).^43^ Other patient factors such as current or recent chemotherapy treatment, prior radiotherapy treatment, iron deficiency anemia, osteopaenia, and patient age can all influence the appearance and cellularity of the background bone marrow.

Peritoneal, retroperitoneal, GI, and GU tract disease are characteristic sites for mILC with the peritoneum involved in up to 93% of cases in autopsy studies and, as with bone metastases, can be underestimated or occult on CT.^6^ Abdominal mILC presents late and is associated with hydronephrosis, bowel obstruction, and poorer overall survival.^13^^,^^14^ A lobular histology has been found to be an independent risk factor for peritoneal spread in breast cancer, however the sensitivity of CT and PET-CT in detecting peritoneal disease varies widely according to size, site, and tumor subtype.^44^ Recent studies have shown that peritoneal and retroperitoneal metastases from ILC are common additional sites of disease seen on WBMRI but not identified on conventional imaging.^18^ A recent large retrospective study evaluated the performance of CT vs WBMRI in the detection of peritoneal disease in colorectal cancer using histology as a reference standard. The sensitivity, specificity, PPV, and NPV for peritoneal disease detection for CT was found to be 43.2%, 95.6%, 84.5%, and 75.2%, respectively, whereas for WBMRI it was 97.8%, 93.2%, 88.9%, and 98.7%. WBMRI has a far greater sensitivity than CT for detecting inoperable disease (*P* < 0.0001).^45^ Similar results were observed in an earlier study assessing peritoneal disease spread in ovarian cancer.^46^ GI tract involvement in mILC is diffuse and infiltrative and involves the submucosa first. Linitus plastica occurs when mILC involves all layers of the stomach which appears small and diffusely thickened and often occurs in the presence of other sites of peritoneal disease.^47^^,^^48^ MRI outperforms CT in identifying the bowel wall layers and tumor infiltration (Figure 5).^49^ 

Liver involvement is observed in 43%-68% of mILC cases and can be discrete or diffuse the latter often being missed on CT.^7^ The retrospective review from Bhaludin et al in 2022 comparing WBMRI to CT, BS, and ^18^F-FDG PET-CT found that WMBRI detects additional sites of disease not visible on conventional imaging with the most common site being the liver (34%).^16^ In the retrospective study from Kosmin et al evaluating the addition of WBMRI to CT in treatment decisions for patients with metastatic breast cancer, in the cases where visceral or soft tissue disease were reported only on WBMRI the detection of disease within the liver was the most common “discordant” site.^19^ Liver pseudocirrhosis is the development of fibrosis, nodularity, parenchymal atrophy, caudate lobe hypertrophy, and capsular retraction seen in the context of metastatic breast cancer either due to the presence of infiltrative liver disease or as a response to chemotherapy.^50^^,^^51^ Distinguishing active liver mILC from post-treatment fibrosis in the presence of pseudocirrhosis on conventional imaging is often very challenging and can be either under or over estimated.^16^

Triple negative and HER2 positive tumors are more commonly associated with brain metastases than luminal hormone receptor positive ILC and IBC-NST tumors.^52^ FLAIR sequences through the brain are used as a screening tool within the WBMRI protocol but separate post-contrast images of the brain can be performed at the time of WBMRI if there are symptoms that might suggest metastatic disease or to further evaluate equivocal findings picked up on the FLAIR sequences.

Solid pleural disease in mILC is less common than in mIBC-NST but can be detected by MRI at a relatively small size.^36^ A systemic review and meta-analysis from Cavion et al found a per-lesion sensitivity of MRI for nodules 4 mm or larger in a cohort of 1354 patients was 87.7% and subgroup analysis demonstrated that MRI sensitivity was 98.5% for nodules measuring at least 8-10 mm.^53^ CT thorax can be considered in addition to WBMRI and a non-contrast CT thorax is sometimes performed with WBMRI at baseline if there is suspicion of lung or pleural involvement.

Radionuclide BSs using Tc99m-methylene diphosphonate (MDP) are still variably used in the UK and Europe even though it has been found that the false negative rate in omitting BS with CT at staging breast cancer is less than 1%.^54^^,^^55^ They are however standardized, validated, and variably incorporated into clinical practice. The uptake of Tc 99m radiotracers is a nonspecific indication of osteoblastic activity leading to false positive cases in the presence of a broad range of conditions such as Paget’s disease, benign fractures, degenerative joint conditions, and inflammation. The use of BS for response assessment in bone metastases in breast cancer is limited due to the “flare” phenomenon in which an increase in sclerotic extent or the emergence of new lesions may be precipitated by a treatment response and can often be misinterpreted as disease progression.^56^ BSs are not recommended for use in the assessment of response within current UK and European guidelines and at present there is no recognized imaging criterion for the use of BS in metastatic breast cancer.^38^

Fluorodeoxyglucose (FDG) positron emission tomography-CT (^18^F-FDG PET-CT) is recommended as first line for staging in inflammatory breast cancers and indicated in problem solving when other imaging modalities are indeterminate. The UK (RCR), European, and international guidelines are now recommending ^18^F-FDG PET-CT for staging all stage III breast cancers but not universally for all tumor subtypes.^57^^,^^58^ The latest ESMO guidelines for advanced breast cancer recommends that ^18^F-FDG PET-CT should not be used in lobular breast cancer assessment and the most recent PET-CT guidelines in the UK have indicated that ^18^F-FDG PET-CT is “less useful” in lobular subtypes. It is recognized that ILC metastases are typically hypometabolic and the FDG avidity seen both within the breast primary and metastatic sites are lower in lobular breast cancer than other subtypes.^57^ This is thought to be related to a combination of a lower grade and cell density, the infiltrative nature of lobular cancers, and a lower level of GLUT-1 expression (Figure 6). Sclerotic skeletal metastases more commonly seen in lobular subtypes are also known to be less metabolically active than lytic metastases more often seen in other subtypes. In a retrospective analysis of patients with newly diagnosed stage III breast cancers, ^18^F-FDG PET-CT upstaged disease to stage IV in 22% of IBC-NST subtypes but in only 8% of ILC subtypes. All IBC-NST tumors were FDG-avid however 25% of lobular metastases were not.^57^ Retroperitoneal and GU disease in addition can be masked by urinary FDG uptake (Figure 7). 

New tracers are currently being evaluated and aim to overcome some of the limitations of imaging with ^18^F-FDG. FES (^18^F-fluoroestradiol) PET-CT uses a tracer that detects ERs and although not yet in clinical use in the UK, is FDA approved in the US for use in patients with ER+ disease, and has been incorporated into the latest NCCN guidelines for consideration in patients with a lobular histology.^40^ A small (*n* = 17) pilot study using FES found abnormal uptake in 88% of lobular tumor sites and resulted in a stage change in 18% of cases when compared to ^18^F-FDG.^59^ A phase 2 trial is underway in the UK comparing ^18^F-FDG with ^18^F-FES-PET-CT (NCT 06557148) but this is not due for completion until 2027. FES-PET-CT has also been found to be useful in cases where ER status is in question or where biopsy of tissue is challenging and can assess ER profile across all tumor sites to guide appropriate treatment. False negative studies can occur for patients using ER blocking drugs however and long washout periods are required prior to scanning with FES-PET-CT which can take up to 28 weeks with for patients stopping fulvestrant.^60^ Gallium labeled fibroblast activation inhibitor (FAPI) PET-CT has also been suggested as an alternative tracer to ^18^F-FDG in the evaluation of extent of mILC although the evidence base is limited. In a small retrospective study, 23 women diagnosed with ILC underwent both ^18^F-FDG and ^68^Ga-FAPI PET-CT. ^68^Ga-FAPI PET-CT demonstrated a statistically significantly higher tumor activity and higher tumor to background uptake ratios (*P* < 0.001) than ^18^F-FDG PET-CT and ^68^Ga-FAPI PET-CT identified more metastatic lesions.^61^

## Whole-body MRI—advanced techniques and future directions

Advances in coil technology and the introduction of parallel imaging are already helping to reduce scan time and increase image quality by increasing the signal to noise ratio. Artificial intelligence (AI) acceleration techniques and deep learning (DL) algorithms such as Deep Resolve and Air Recon are further improving image quality scan times.^62^ ADC and rFF measurements have been used as surrogate biomarkers of response. Measurements such as global ADC (gADC) and total diffusion volume (tDV) are segmentation techniques that can be used as a visual guide to assess response without relying on individual tumor lesion size change.^63^ Machine learning (ML) can help with automatic lesion detection and segmentation; the MALIMAR and MALIBO studies have developed a machine learning method with the aim to reduce the radiology reading time for staging WBMRIs.^64^^,^^65^

## Discussion

At our institution, WBMRI is currently used for the staging of all lobular breast cancers. It is also used for response assessment for patients with bone-only or bone-predominant disease, when CT findings are equivocal or when there is an unexplained tumor marker rise. Although WBMRI is a standard imaging technique in the management of tumors such as multiple myeloma and prostate cancer, prospective data supporting its use in breast cancer are limited. Guidelines have been published for the use of WBMRI for Myeloma (MY-Rads), Prostate cancer (MET-Rads-P), and in screening for high-risk groups such as those with Li-Fraumeni syndrome (ONCO-RADS) but no such consensus recommendations exist yet for breast cancer.^26–28^ These established guidelines have standardized the imaging protocols, the quality of the images acquired, and the reports produced in those clinical settings. Retrospective studies have found that WBMRI is a more accurate test in detecting mILC and monitoring response to treatment than the currently recommended CT, BS, and ^18^F-FDG PET-CT however these study populations are small and from single centers often involving mixed metastatic breast cancer populations rather than dedicated mILC cohorts. The only prospective study at the time of writing comparing WBMRI to CT in the evaluation of metastatic breast cancer included only 8 patients with a lobular tumor subtype.

Metastatic ILC is often underestimated or equivocal and sometimes occult on conventional imaging techniques. WBMRI can identify a greater extent of metastatic disease potentially preventing late presentation with complications of disease infiltration and enabling earlier detection of PD, thereby influencing patient management decisions. Emerging evidence suggests that earlier detection of PD in bone may reduce skeletal related events and hospitalization.^66^^,^^67^ There are currently no published data however demonstrating that the improved detection of metastatic disease with WBMRI translates to measurable patient outcomes, such as PFS, OS, or quality of life. WBMRI does not use ionizing radiation, does not require IV cannulation, and allows assessment of FDG negative disease. WBMRI shows potential for the development of criteria assessing response in bone metastases currently lacking for both CT and BS. In addition, WBMRI allows prompt detection of complications such as fractures, malignant spinal cord compression, earlier detection of liver, retroperitoneal, and peritoneal metastases and therefore reduces the need for additional imaging. The incidence of lobular breast cancer is rising and the treatment options available for this heterogeneous patient group are becoming more personalized. As imaging appearances become increasingly complex it has never been more important to accurately stage and assess response in this patient cohort and WBMRI has been found to be more accurate in the response assessment in patients who have had multiple lines of treatment. Interobserver agreement studies using WBMRI in breast cancer are very limited and whilst results have documented favorably high Kappa scores there are no studies looking at a pure mILC cohorts.^68^ Interobserver agreement is dependent on data from other tumor types but has shown that the level of agreement when interpreting WBMRI improves amongst experienced readers.^69^ It may be that the interobserver variability gap between expert readers and those less experienced is wider for mILC than IBC-NST given the often subtle and infiltrative patterns that are characteristic of mILC but this is yet to be established. It should however be recognized that there is a learning curve when starting to report WBMRI, even for the experienced cross-section reporting radiologist and interobserver agreement statistics should be considered when setting up WBMRI studies for mILC cohorts.

Challenges for the widespread adoption of WBMRI in mILC include the lack of standardized protocols, validated reproducibility data and response criteria, patient time on the scanner, and lack of staff training. Health economic evaluations of WBMRI compared with CT, BS, and PET-CT should also be prioritized. Communities such as the Quantitative Medical Imaging Coalition are providing frameworks for standardization of WBMRI techniques across different vendors and magnet strengths and both education and dissemination of information for those wishing to set up a local service. WBMRI protocol development within clinical trials of patients with early or mILC may also serve as an important platform to facilitate adoption of this promising modality.
